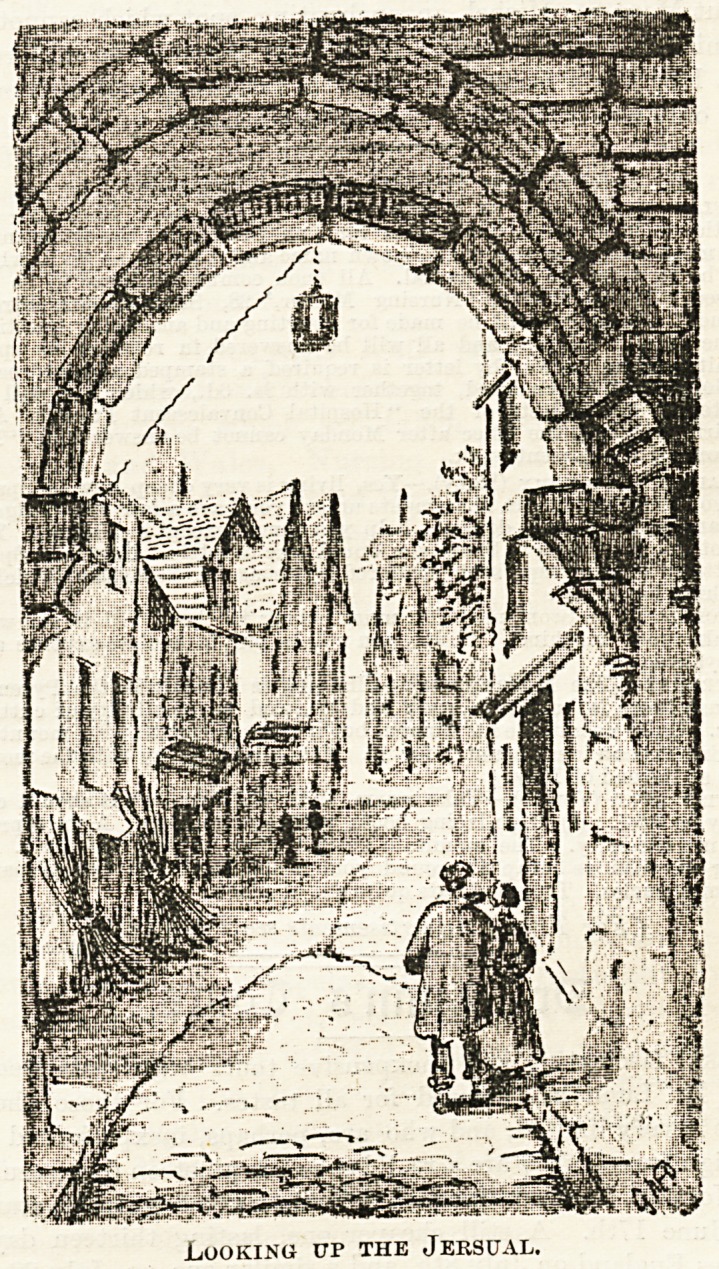# "The Hospital" Nursing Mirror

**Published:** 1899-05-06

**Authors:** 


### "The Hospital" Nursing Mirror.

Being the Nursing Section of "The Hospital."

[Contributions for this Section of "The Hospital" should be addressed to the Editor, The Hospital, 28 & 29, Southampton Street, Strand, London, W.C., and should have the word "Nursing" plainly written in left-hand top corner of the envelope.]

### Notes on News from the Nursing World.

### THE CENTRAL BRITISH RED CROSS COMMITTEE.

The result of the conference between the representatives of the National Society for Aid to the Sick and Wounded in War, the St. John Ambulance Association, and the Army Nursing Reserve, who were invited by the Secretary for War to meet the heads of the Army Medical Service and discuss the lines upon which a central organisation for bringing voluntary aid throughout the British Empire into touch with army medical requirements might be created, is distinctly a matter for congratulation. The formation of a central British Red Cross Committee, recognised by the War Office as the official channel through which offers of voluntary aid in time of war will be accepted, is just what was necessary. Nor could the Army Nursing Reserve be better represented on the committee than by H.R.H. Princess Christian and Miss Wedgwood. The representatives of the other societies have also been admirably chosen. Lord Wantage is chairman of the committee, who at present hold their meetings in the board-room of the Medical Division of the War Office, 18, Victoria Street, Westminster.

### A REGISTER FOR NURSES IN IRELAND.

The Local Government Board for Ireland give notice to trained medical and surgical nurses that they are about to open and keep a register for trained nurses, and that a certificate as to the fact of a nurse's name being entered in the register will be regarded by them as sufficient evidence of her professional qualifications for the post of any trained nurse in any Poor Law union, infirmary, or district hospital. On application to the Secretary of the Board at Dublin any trained nurse on their register will receive notification of the particulars of each appointment that falls vacant.

### THE RECEPTION OF NURSES BY THE LORD MAYOR OF BIRMINGHAM.

It was a happy idea on the part of the Lord Mayor and Lady Mayoress of Birmingham to prepare an evening's entertainment on April 25th for the members of the nursing profession who are engaged in and about the city. To the reception at the Council House about 650 nurses had been invited, and there were asked to meet them the members of the City Council, the chairmen and secretaries of the various hospitals and institutions, and a few private friends of the Lord Mayor and Lady Mayoress. The staffs of thirteen hospitals, six district nursing societies, and a number of training institutions and nurses' homes were the recipients of invitations. It was, in comparison with some which had been held, a small party, but the Council House was as brilliantly arranged as if the occasion had been one of the great social gatherings which annually take place. The impossibility of closing hospitals, or leaving them unattended, made requisite the division of the evening's entertainment into two portions. To the first came one-half of the nursing staff, and these having gone through the programme, returned to relieve their sisters who had arranged to come later. The entertainment was therefore necessarily of a simple nature. During the presentations Mr. A. W. Gilmer's Bijou Orchestra played musical selections. The guests passed on to the Council Chamber, where Mr. Douglas Beaufort gave an exhibition of pure sleight-of-hand, and musical and ventriloquial sketches. The audience was one of the most picturesque which has ever filled the Council Chamber. By request the nurses wore uniform. Grey dresses and snowy-white caps and aprons mingled with the pink costume of the nurses from the workhouse infirmary, and here and there the colours of a lady's ordinary evening dress presented a striking effect. When Mr. Beaufort finished there was an interval, during which the guests had an opportunity of wandering round the Art Gallery, where refreshments were served. For the late-comers at half-past nine exactly similar proceedings were arranged.

### THE PERMANENT HOME FOR KENSINGTON NURSES.

It is satisfactory to learn that the movement for the erection of a new home for the nurses of the Kensington District Association for Nursing the Sick Poor in their own Homes is making progress. A certain amount of money has been actually raised towards the £2,500 for the purchase of the lease or freehold of a house in a central part of Kensington. Quite recently H.R.H. Princess Louise has been over the existing home, and pronounced it to be altogether inadequate for the purpose. The matter is primarily one for the inhabitants of Kensington. They best know the value of the work done by the trained ladies who belong to the Association. Last year they nursed 854 cases, as compared with 798 in 1897, and paid 19,564 visits. Their duties are onerous, and are often performed in an infected atmosphere. It is only right that their home should be comfortable and capacious, and we cannot doubt that the wealthy people in the Court suburb will soon furnish them with the accommodation which they require, alike in their own interests and in those of their patients.

### THE NOTTINGHAM AND NOTTS NURSING ASSOCIATION.

At the meeting of this association in Nottingham last week the twenty-third annual report was adopted. The most salient point is that during the twelve months the district nurses attended 880 patients and paid 31,485 visits. Of this number of sick persons 200 were being attended by the staff of the General Dispensary, who have frequently expressed their appreciation of the help which the nurses afforded. No less than 270 applications were received directly from the poor themselves. In seconding the adoption of the report Mr. Birkin spoke in moving terms of the comfort and assistance the large number of visits had given, and said that none but the beneficiaries themselves could tell how many lives had been saved by their watchful care. He insisted that the day of trial and experiment of these nursing institutions had passed, and that now "almost every town and county in the country were speaking eloquently in their praise."

### THE DENOMINATIONAL QUESTION AT DUBLIN.

ON Thursday and Friday last week a sale of work was held in Dublin on behalf of the Nurses' Home attached to Steeven's Hospital. The Roman Catholic press of Dublin speak in the warmest terms of the work done by the nurses, and describe the hospital as "conferring priceless advantage on the sick and injured poor." But they protest that the institution is inaccurately entitled "strictly undenominational." It is alleged that not a single Roman Catholic is on the medical staff, and that "when a Roman Catholic surgeon, one of the ablest in the city, offered himself some little time ago for election to it, the Protestants rallied all their forces on the Board of Governors to defeat him." There is, of course, no reason why Protestants, as well as Roman Catholics, should not found, and carry on, hospitals for their co-religionists at their own expense—though the fewer of charitable institutions on these lines the better—but, no matter how splendid the work achieved, the public generally who are invited to support them by subscriptions should not be told in a printed appeal that they are "strictly undenominational" if they are not so.

### NURSES AT THE ROYAL FREE HOSPITAL.

NOT the least urgent of the needs of the valuable institution in Gray's Inn Road is better accommodation for the nursing staff. At the Mansion House meeting last week Mr. Charles Burt drew particular attention to the total inadequacy of the existing sleeping and sitting-rooms. The public, we are sure, will agree that, while it is essential to make improvements in the wards for the benefit of the sick and suffering poor who obtain admission to the hospital "without money and without price," it is likewise of great importance that those who nurse them should be able, when they are off duty, to obtain the sleep they so much want under healthy conditions, and not be cramped for space in the sitting-rooms.

### NURSING IN ROME.

IN the recent notice of the proposed Anglo-American Nursing Home in Rome a correspondent thinks we overlooked the fact that for six years an English order of nurses at 45, Via Castelfidardo, have received into their home English-speaking patients of all ranks. These nurses, she mentions, have been trained in our best London hospitals, and having herself been a patient at their home she is able to speak personally as to its comfort, good nursing, and "the unbounded kindness of the nurses to all sorts and conditions of people." Our correspondent adds: "During the last few years the nurses have received 68 English people into the home—they admit Italians also—and are constantly refusing applications for which they have no room." While rejoicing at the excellent work done by the ladies referred to, we may cite the last sentence of our correspondent's letter as strongly confirming the need for the new nursing home. The fact that they have "constantly" to refuse applications from people desiring admission does but emphasise the complaint that more skilled nurses and more accommodation for patients are badly wanted in Rome.

### ON BEHALF OF THE CHILDREN.

THE annual competitions of the Children's Salon, an organisation originated by the *Gentlewoman* for the youthful readers of the paper, with the object of bringing them in touch with "the children of the poor," were held on Saturday afternoon at the Westminster Town Hall. The idea is to encourage young folks "to exercise their talents in art, literature, music, and work, for which prizes are awarded, with the special object of endowing cots in children's hospitals." Already the money collected has endowed in perpetuity two cots, one at the Victoria Hospital for Children, and the second at the North-West London Hospital, whilst a helping hand has been given to several other charities. This year the money will go towards the endowment of a third cot in the North-Eastern Hospital for Children, Hackney. H.R.H. Princess Christian is one of the patrons. On Saturday more than a hundred competitors entered their names, and among the judges were Miss Ella Russell, Herr Johannes Wolff, Mr. William Ganz, Mrs. Brown Potter, Mr. Franklyn M'Leay, Mrs. L. T. Meade, and Mr. Anthony Hope. The prizes were presented by "Levana," the presiding genius of the "Salon." There were a great many visitors, and the endowment fund must have profited considerably.

### THE HARRISON CHARITY.

THE new scheme for the charity of John Harrison includes one feature of interest to the nursing world. A sum of not more than £200 a year is to be given in subscriptions of not more than £75 a year to the Leeds Nursing Association, the Huddersfield Victoria Nurses' Association, the Dewsbury Sick Nursing Association, or other nursing institutions in Leeds, Halifax, or Dewsbury, "provided that agreements have been made with each of the institutions receiving subscriptions, by which they undertake to provide gratuitous nursing to those benefiting under the trust when sick or injured." This is a very proper condition, because the income of the property is to be expended solely for the benefit of those descendants of a sister of John Harrison whose names shall be on the trust register, "and who are in need fo charitable relief."

### SHORT ITEMS.

H.R.H. THE DUCHESS OF ALBANY opened a grand bazaar on Wednesday afternoon at the Portman Rooms, in aid of the Great Northern Central Hospital. There was a large attendance, and the stalls were most admirably arranged.—The usual monthly lecture at the Trained Nurses' Club, 12, Buckingham Street, W.C., will be on the 26th inst. at a quarter to eight p.m. The lecture is Miss L. Appel, M.B., B.S., B.Sc.Lond., and the subject, "The Peritoneum in Health and Disease." Members and friends of the club who have kindly recollections of Mrs. Nichol, secretary for so many years to the Midwives' Institute and Trained Nurses' Club since its foundation in 1886 till within the last two years, will regret to hear of her death which took place on Easter Day, after long ill-health.—Miss E. B. G. Gray has resigned the post of superintendent of the Metropolitan Nursing Association for Providing Trained Nurses for the Sick Poor, and is leaving at the end of three months. Miss Gray has been connected with the association for seven years—from 1892 to 1895 as probationers' staff nurse, and senior nurse successively; and from 1895 to the present date as superintendent.

### Antiseptics and Operations.

A Lecture delivered to the Nurses of the Victoria Hospital, Hull, by Alf[illegible] Parkin, M.S., M.D. (Lond.), Senior Surgeon to the Hospital.

(*Continued from page* 61.)

Now say a patient is going into the operating-room. First wrap him up well. As a rule he is timid and nervous, and it is not very nice to be taken out of bed, carried along a corridor, and then put down on a cold operating table. And remember that if a patient is going to have his feet operated upon it is not necessary to take everything off his chest; if his chest is to be operated upon, then there is no need to take his stockings off. Patients are very apt to take chills on the operating table; one I know of got pneumonia in that way. So don't forget to wrap up your patient well, and, so far as you can, keep him wrapped up whilst in the operating-room.

Now the patient is in the operating-room, so let us next consider what it is wise to have in an operating-room. All sorts of things happen in an operating-room, so it must be made so that you can wash it down quickly and easily. Most operating theatres are made with washable walls and floors, and the best thing for these washable walls and floors is white tiles. Of course, they are very cold, especially in winter, but this can be remedied by having a larger fire and an efficient hot-water apparatus. There should be as little furniture as possible in the room, because furniture gathers germs, or as you call it, dust. Then you want a good light, plenty of hot and cold water, plenty of clean towels, sponges, and a good supply of basins and mackintoshes. If you are preparing for an operation in a private house, try to get ready all these things to the best of your ability. It is no use only teaching you what you require in a hospital, because you will have to adapt yourselves to the work in a private house as well as to that in a hospital.

And now we must elaborate a few of these things. The hot and cold water here is, of course, supplied on the premises. In a private house you have to get your own hot water, and after you *have* boiled and cooled it, do not stir it up with a dirty hand to see if it is sufficiently cool; the mere stirring of the water with the hand that is not quite clean is sufficient to make it poisonous. What irritates me extremely is to see a nurse draw hot water from a tap in the theatre here, and then put her poisonous hand in to see if is quite warm! In a private house one frequently comes across a nurse who pours water into basins that are not quite clean, and when she has filled her basins she makes matters ten times worse by putting her hands in. Do not forget you can tell from the outside of the basin as well as from the inside whether or no a lotion is of the proper temperature.

Clean towels are the next important feature. When anything important has to be done, I always like a couple of clean towels that have been boiled for half-an-hour with a little soda to place around the site of the operation, because they are absolutely pure, and you can put instruments on them without fear of infecting your patient. When you have boiled them, do not wring them out with your hands; put them in another clean towel and screw them up as you would a hot-water compress.

And now we come to instruments. You know that in the operating theatre you have the instruments to look after, whilst in private the doctor brings his own, and is responsible for their cleanliness. As you know, we always boil our instruments before an operation and after. Boiling water will kill any number of germs, and so if you boil instruments or towels you may be sure there are no germs in them. They are boiled in hot water containing a little soda, the soda being simply to prevent the instruments from rusting. The water here is very hard, and steel instruments rust very quickly.

But do not depend on boiling only. When you take dirty instruments away, you say, perhaps, "Oh! yes, we will boil them well," and straightway put them into the boiler. That is not enough; brush all the crevices well before you put them into the hot-water boiler; and while on the subject of instruments, I may as well tell you that before you begin to clean any instruments after an operation you should first take out the knives and needles. If you throw them into a tray or into a boiler, you will find the knives are absolutely useless and your needles hopelessly blunt; so first wrap up the blades of the knives in lint, and stick the needles into another piece, before putting them into the steriliser.

Another important feature in an operating room is that you must see that everything is ready for the anæsthetist. The anæsthetist requires, of course, a bottle of chloroform, and a bottle of ether, a small quantity of brandy and water to inject in case the patient suffers from the chloroform (equal parts of brandy and water are best), and then do not forget tongue forceps, which you have no doubt often seen on the table, and a hypodermic syringe. I think any anæsthetist at this hospital has just cause of complaint if he does not find these things ready on the table for his use.

Well, now, what does a nurse do in an operating theatre? The main duties of the nurse are to prepare the instruments, get ready the mackintoshes and boiled towels, prepare the sponges, and, when required, to produce the dressings. The sponges we use at the hospital are pieces of boiled lint. We have done away with the ordinary sponges, though it is only within the last few years that they have been discarded. They are nicer for the operator to use, but are very difficult to clean perfectly after use, *i.e.*, to render free from germs. We now use lint which has been boiled for an hour in water containing a little soda, after which it is stored in carbolic acid (1 in 20) to keep germs away from it until it is wanted, and it should not be touched by the hands of anyone, except those of the operator. The nurse must not put her hand into the jar or basin to get it out. In all probability she has been putting a blanket on the patient, or putting him on the table, or a thousand and one things which may have soiled her hands with germs. She must get out the lint with a pair of forceps, which lay ready near the jar, and put it direct into the basin of lotion.

It simplifies matters a good deal when one uses sponges of this kind, because the lint is simply thrown away after use and requires no further looking after, whereas if sponges at 3d. or 4d. each are used, they have to be cleaned, and you have to clean them particularly to make sure they are all right.

When the operator comes, he puts on an apron, and you will then notice how careful he is to wash his own hands, and after he has washed them you will see he touches nothing but the patient. If you keep your eyes open in our operating theatre, you will see you are not the only persons required to take proper precautions. Watch the surgeons and follow their example.

The surgeon then proceeds to do his operation, and after he has finished it, he cleans everything about the wound and applies the dressings. And now we refer to the original point —why does he apply dressings? Simply to keep out germs. The dressings do not heal the patient, any more than the strapping applied to a cut finger heals the cut. It is a popular fallacy that the application of a bit of strapping causes the wound to heal, but it does nothing of the kind, it simply keeps the edges of the wound together whilst Nature does the healing. Similarly dressings are applied to cover up a raw surface or a wound to prevent germs getting access to the wound, until Nature can set to work to heal it. If germs get to the wound, you are certain to get some form of suppuration. So the object of the dressings is to prevent the wound getting infected—or, in other words, the dressings are antiseptic.

Now, as opposed to antiseptic (and it is a word which you will sometimes come across), is aseptic. Antiseptic means anything which prevents poisoning—aseptic means "non-poisonous," the meaning of which you will see in a few minutes. Now, I have all sorts of dressings here, which I got down for you to see, so that you may know and recognise them at a future date. For it is no use you thinking that the only dressings used are those you have here in the hospital. All these dressings here have some antiseptic powders in them; there are some among that you have perhaps not heard of before. There is the usual mercury and zinc cyanide gauze. Besides this there are carbolic gauze and wool, perchloride of mercury gauze and wool, and so on. All these various sorts of gauze and wool are used; the gauze and wool are identical in all, but the antiseptic employed is different in each case.

Aseptic dressings simply mean gauzes, lint, or cotton-wool that are quite free from germs. At some hospitals you will be told to use nothing but aseptic gauzes; it comes to very much the same thing as using lint which has been boiled to kill the germs in it. If germs get to aseptic gauze they won't get killed. The distinction between the terms "antiseptic" and "aseptic" is one that you will probably have a little difficulty in remembering; indeed, one very often hears nurses and others confuse the terms.

Well, you take care your dressings are ready when wanted at an operation, and when they have been applied, you take your patient back to bed, keeping a careful watch on him for fear sickness comes on. The after-treatment of a patient following on an operation is, as a rule, very simple. An operation is a thing that very frequently, especially in children, leads to a good deal of shock; the patient is cold, chilly, with a pale face and quick pulse. Have hot-water bottles ready and try to get him warm again; wrap him up in a hot blanket, and, if ordered to do so, give a little brandy, or, in some cases, inject into the rectum half-a-pint or more of hot water with a little brandy, but you are not to do this, or, in fact, to give the patient anything at all, on your own responsibility. Take care that the hot-water bottles are ready at the close of the operation, and the same with the hot blanket—take care it is quite ready, too. When the patient is cold and collapsed, you cannot get a blanket ready for him in a minute; and while you are preparing it he may become still more collapsed, or may die.

Think of all these things and have them ready beforehand; and if you cultivate habits of this sort one will feel gratified that the lecture has not been thrown away. It is no good having to set to work afterwards to do things which have been forgotten, when the very omission at the time might seriously endanger the life of a patient or possibly retard his recovery.

### Where to Go.

St. James's Hall.—Joseph Wieniawski will give a concert of pianoforte and chamber music, assisted by Herr Theodore Werner, on Thursday, May 11th, at three o'clock. Tickets at the hall.

Dowdeswell Galleries, 160, Bond Street.—An exhibition of pictures illustrating passages from British poets, by Byam Shaw, is being held at the above galleries.

Thursday, May 11th.—Concert at St. James's Hall at 8.30 p.m. by the Magpie Madrigal Society, in aid of the new Hospital for Women, 144, Euston Road.

### Typical Patients.

### THE KNOWING PATIENT.

It was rather late on Saturday night when he arrived. The lights were down and most of the patients were asleep, when the door opened and the bright light of the outer lobby streamed down the ward. As the porters wheeled him in, the new patient gazed round him with a fatuous smile. He was evidently in the condition that is described in the East End as "a bit jolly-like, you know." "Well, nurse," he said, jovially, as she came forward, "it's a tib-and-fib this time, I believe. Last time I was in this 'ospital, it was a fractured patella." He rolled out the phrase with evident pride. "That was a nine weeks' job," he continued. "I know all about 'ospitals, I do. There ain't much you can teach me about 'em."

So it seemed. And when he announced next day that his wife had been a nurse, his triumph was complete. There is no more trying patient than the man whose wife, or sister, or ever so distant relation has been a nurse, and the new arrival was no exception to the rule.

His former residence in hospital, combined perhaps with his wife's professional experience, had provided him with a few medical terms which he used more or less correctly as occasion offered. When temperatures were being taken he would inquire affably, "Any *py*-rexia, nurse?" with a marked emphasis on the *py*.

Temperature-taking, indeed, seemed his *forte*. He looked forward to his convalescence when he could help the nurses by taking the temperatures of the ward. He knew exactly how it was done, his wife having been a nurse. In fact, what he did not know about temperature wasn't worth knowing. Then he would try to draw his nurses into a discussion on thermometers, charts, &c. Failing that, he would discuss the subject *ad nauseam* with the patients, throwing out remarks that there were "nurses *and* nurses," with obvious application. His fellow-patients listened with a certain amount of admiration to begin with, but that faded away; and on the rare occasions when he seemed snubbed for the moment their joy was great.

On boiled mutton days he would examine his plate with the air of a scientific investigator, and then, while his face lit up with a smile, would exclaim, "Why, it's mutton! blest if I didn't think it was game. But then things are so well cooked in this 'ospital, a man don't know 'alf the time wot he is eatin'!"

On one occasion the visiting surgeon stood at his bedside surrounded by a crowd of dressers, and our friend looked up at the great man with a pleasant and even patronising smile. "It's in good position, ain't it, sir," he said, with an explanatory wave of the hand towards his leg. "I was feelin' it this morning, and it seemed to me it was growin' *callous* already, as you might say." There was a spontaneous burst of laughter from the students, and the patient was sufficiently discomfited to take the subsequent rebuke of the Sister very quietly.

At last the day came when he was able to get out of bed and hobble about on crutches. As might be expected, he refused all offers of assistance in his first attempts at walking. "There ain't much you can teach me about crutches," he began. "Last time when I fractured my patella"— "Pity it worn't your jaw," interrupted an exasperated listener, and the ward grinned in sympathy. He turned round hastily, and, twisting his crutch, fell heavily to the ground. A chorus came from the beds, "There ain't much you can teach me about crutches," but the only answer was a groan.

It was a fractured femur this time.

### The Nursing Question in Italy.

During the last ten years or so there has been a distinct movement in the Italian nursing world. A far greater one, in fact, than is known even to the people most interested in it. For we have here but another proof of the truth of the saying, "It was not Christopher Columbus who discovered America, he merely first landed there; others had long before known of its existence." Whoever may be the Christopher Columbus of trained nursing, receiving recognition as its discoverer, will owe her success largely to the quiet work of her predecessors, who already are convinced of its "existence" and are aiming at its realisation.

The little knowledge, however, that exists of this movement is probably due to its being—like Italy herself—not exactly "one and united," but a series of disjointed efforts, even sometimes of petty rivalries, with no organised scheme of action or recognised standard. Each hospital has its different system of training, and each town has its different societies, which, in imitation of the original "Red Cross," profess to train, and, as a matter of fact, supply "nurses" of varying degrees of competence. Unfortunately, however, they have all failed to understand the absolute necessity of three conditions, namely, that (*a*) a nurse can only be thoroughly trained in the wards of a general hospital; (*b*) this training must continue for a sufficiently long period to give opportunity for wide experience and practice; (*c*) the training must be under the direction and actual instruction of a woman who is herself a trained nurse. Some of these societies train their nurses only in the out-patient departments of hospitals; others obtain permission for their pupils to work for three months in some hospital ward; others do even less and attempt to train solely by lectures. Some have realised the necessity of combining ward practice and theoretical lectures, but nowhere have they understood that thorough training needs also the intervention of a nurse as superintendent or *direttrice*.

Looking broadly at the matter, there seems very little doubt that the real reason for the small result of the many efforts made these last ten years is due to the fact that philanthropic ladies and doctors have attempted the whole training of nurses themselves. Not only have all the nursing societies dispensed with having trained nurses on their committees (the Italian trained nurse has only just come into existence, it is true), but hospital administrations have dispensed with their co-operation in the organising and instructing of the nursing staff.

The head of the hospital nurses is always a *direttore* or *soprintendente* (doctor or professor). It is he, and not the Mother Superior, who rules their lessons, regulates their duties, orders their uniform, and fines them for slatternly appearance, &c., &c. What is more, he does it admirably, as far as he goes. And though it seems a digression, it is necessary to remark that the Italian man is naturally more gifted with domestic talent than the English one. Amongst the virtues of the Italians is a much greater patience and power of entering into small details on the part of the man in the middle and lower classes. It is the father, as much as the mother, who amuses the baby in most working homes; on fête days it is he who carries the little ones, or leads them by the hand, the mother accompanying or not, as cooking exigencies permit or forbid. Even in the upper classes the father occupies himself constantly in the nursery arrangements, advising in matters of dosing and dieting, as in those of education and amusement.

Therefore, it is a fact that Italian doctors are infinitely more capable than English ones of doing what is commonly considered woman's work. But still they cannot do it as a woman could; it is waste of time and brains that they should attempt it, and in the end they must fail, for what is really wanted is example. A doctor cannot become a nurse, night in, night out; day in, day out! Consequently, he cannot inspire the enthusiasm, the sense of *esprit de corps*, of emulation, which a woman who is herself a nurse inspires. For she has herself done all that she expects the newest probationer to do, and can demonstrate how best to do it; she can show them all that a nurse should aim at, all that she can be to her patients. She can understand, as no man can, the difficulties, discouragements, and dangers, as well as the joys, satisfactions, and blessedness of a nurse's life, for she has herself experienced them. She also, being always present in the wards (in the person of the head nurses), can organise and simplify the work and regulate economies in a way that no doctor, however constantly appearing there, could do; whilst, finally, she only can give the home atmosphere, the quiet and comfort, the privileges in the midst of restrictions, which make all Italians who visit an English hospital exclaim, "We have hospitals, but you have homes for your sick."

Why it is that the Mother Superior is not granted the power which would enable her to be all this to her hospital is a question too complicated to be here discussed. But it is doubtless largely due to the fact that she has not had the training which an English or American Lady Superintendent must of necessity have had, and which can alone fit her to become the Christopher Columbus of the world of nursing. Whenever such a woman shall appear, doubtless men will recognise her superior fitness for executing much of the work which now lies heavy on their shoulders, and they will willingly entrust her with the better systematising of ward work and the higher training of the nurses, which is alone needed to place their hospitals amongst the highest in Europe.

It is to be hoped that the growing demand in Italy for better trained and more refined nurses for private cases will also help to hasten the movement towards a general higher standard of nursing. Hitherto all nurses who were not nuns were exclusively of the regular servant class, barely able to write and read; kindly as a rule, but utterly unrefined.

During the last four years, however, a remedy for this deficiency has been attempted by opening a school for girls of the small bourgeois class in the general hospitals of Rome, Naples, and Florence. These schools are initiated by, and remain under, the active protection of Roman, Neapolitan, and Florentine ladies and professors. The teaching of the pupils is under the control of a "*direttrice*," who is an American or English trained nurse.

The *direttrice*, beside herself giving lectures and demonstration classes to the pupils, also attends all the lectures given them by the professors, taking notes, and giving repetition lessons on the same. As a result, yearly examinations have been passed most satisfactorily (oral and written) and certificates given; after which the nurse is sent to private cases, living in her own home when not occupied.

The *direttrici* in each town have received eminently satisfactory testimonials from both English and Italian patients regarding he "ability, intelligence, and goodness" displayed by these nurses in the execution of their "pious mission."

The fact that an Italian nurse with English training can now be had in these three towns is really a matter of interest to English residents, or even to those English who may need nursing whilst travelling. Many, from reasons of economy or from objecting to the inconvenience of having English-speaking nurses with Italian servants, may prefer to employ the "native article." Especially as, except for their "newness," these Italian nurses compare by no means unfavourably with the English ones. They come fresh from the best hospitals and clinical wards, where they have aided in the carrying out of all the newest treatments, as well as having received lectures a good way ahead of those to be obtained in most English hospitals.

Added to this, Italian women have proved themselves admirably capable for training as nurses. Their "bed-side diaries" are often more intelligently and accurately kept than those of the more experienced but rather old-fashioned English nurses, which are sometimes found wanting in details of medicine, quantities of nourishment, and systematic division of days (so necessary for the convenience of quick reference by physicians in typhoid cases.)

With regard to the natural attributes so needed in nurses, quickness of perception, tact, absence of awkwardness, &c., Italians possess them largely. They observe everything, and, as proof, one of the Florentines, with her first private case, noticed the very commencement of the Cheyne Stokes respiration, and reported to the doctor, to be verified by him only a day later.

Quietness of voice and manner they have acquired, whilst reticence has been instilled as first code of honour; and consequently no gossiping or repeating of private details relating to their patients has been laid to their charge. Their presence of mind has been frequently proved, both in the operating theatre and in the wards. The English standard of refinement, knowledge of hygiene, and ventilation have been acquired; whilst in unfailing kindness, patience, long endurance, small feeding, and short sleeping they naturally and spontaneously surpass the average of northern nurses.

To obtain one of these nurses in Rome it is necessary to telephone or telegraph to the "Direttrice Infermiera, Ospedale di S. Giovanni"; at Naples, to the "Direttrice della Croce Azzuro, Istitute Suor Orsola, Cariati"; at Florence, "Direttrice Infermiera, Ospedale Anna Meyer." The rules made out by the committees and given to every nurse when called to a case are as follows: The nurse must, on undertaking a case, provide herself with a paper properly filled in by the *direttrice*, and present it to the patient or his family. He or they are begged, when the assistance is finished, to give the nurse back the paper in a closed envelope stating their opinion of her services, and accompanied by her stipend, that she may consign it to the *direzione*. The fee charged for the nurse is five francs for twelve hours' assistance, be it by night or by day. Five francs are also charged for her entire services provided: (1) She is granted seven hours out of the twenty-four for sleeping; (2) she may hear mass on festivals when the condition of her patient permits it; (3) she is to be treated with the consideration due to her mission; (4) does not eat with the servants if possible; (5) has coffee or broth, or something else, when nursing the whole night; (6) may go out for an hour or more a day when the patient's condition allows it.

### The Care and Education of the Feeble-minded.

On Tuesday afternoon Mrs. Dickenson Berry, M.D., gave the first lecture of the course arranged by the Association of National Health Workers at 53, Berners Street, her subject being "The Care and Education of the Feeble-minded."

Mrs. Berry pointed out that in this country the term "feeble-minded" was used to denote that condition hardly to be called idiotcy, but yet showing some mental abnormality. There were many degrees of mental deficiency not to be defined by hard and fast lines, and with them might be associated physical imperfections, arrested development, deformities evident at birth, or of later development. Certain external signs denoted abnormality of brain development—for example, the shape of the skull in the case of hydrocephalic and microcephalic idiots. It might be assumed that if after ten years of age the circumference of the head were less than 18 inches, such a child would almost certainly be imbecile. Mrs. Berry went on to describe more particularly the various types of imbecility and feeble-mindedness, instancing the so-called Mongol type, cretins, &c., with their special characteristics, and explaining some of the causes which led to their development.

Then, said the lecturer, came the question, "What could be done with these unfortunates; could their lot be ameliorated, and could they be made capable of filling a useful place in the world?" Mrs. Berry described the system followed by Seguin, before whose time idiots were looked upon as hopeless of improvement and left to stagnate in their miserable isolation. He believed and taught that all defectives were capable of some training. He trained teachers and founded institutions in France, and his efforts resulted in considerable improvement even in the worst cases. His training followed the lines of education physiological and moral, based upon the theory that the organs and functions reacted one upon the other. Curious cases were quoted in which, by infinite patience, the condition of idiots of the lowest type had been ameliorated by physical exercises and moral influence.

Mrs. Berry spoke of the recently awakened public interest in feeble-minded and idiotic children, and described what has been done in Germany and Scandinavia, and advocated in England by Dr. Shuttleworth, Dr. Warner, and others. In 1892 the Leicester School Board first instituted special classes for the instruction of feeble-minded children, an example followed by the London School Board. Much could be done, Mrs. Berry maintained, by the individual attention possible in these classes, which were limited to a small number, and the teaching carefully adapted. "With these abnormal children in many cases the general vitality was below par; they were of various types, nervous, or dull and heavy, not organically defective, but lacking the brain force to make proper use of their senses. To teach such children, everything must be put before them in concrete form. In the special classes the instruction was adapted to their understanding. They were taught to recognise colours, to do wool work, basket and macrame work, cookery, laundry, and carpentery, such as would help them in later life to support themselves. The success following these attempts had been encouraging; some children had been trained to support themselves almost wholly, others to fill useful places in their homes, but it was estimated that 1 per cent. of the children in the schools are abnormal, and of these a large number must ever remain unfit for ordinary life. Of these, those who have no homes or means of subsistence were of the class that drift into crime and find their way into the workhouses, too often handing down to their descendants the same characteristics and attributes." In conclusion, Mrs. Berry mentioned the Hendon Home for the reception of such cases, founded on the "cottage" system, where the results achieved had been good. It was her conviction that institution life was the worst possible for the feeble-minded, while the colony type of home was undoubtedly the best.

### Presentations.

Miss Jeanie McCosh has been presented with a leather writing case from her fellow-nurses, and a travelling bag from the servants and porters on the occasion of her leaving the City Hospital, Sheffield, after upwards of nine years' work. Miss McCosh carries with her the good wishes of many friends.

### Royal British Nurses' Association.

A quarterly meeting of the general council of the Royal British Nurses' Association was held at the rooms of the Royal Medical Society, Hanover Square, on Friday, April 28th, Sir James Crichton Browne presiding. There was a good attendance of members, several items of interest being on the agenda for discussion.

Miss G. A. Leigh, the secretary, having read the minutes of the previous meeting, the financial report for the year ended March 31st last was presented by the Hon. Treasurer (Mr. John Langton). In accordance, he said, with the suggestion of the auditor, the accounts appeared in a somewhat different and probably more intelligible form than had hitherto been the case. The receipts from members' annual and life subscriptions amounted to £377, an increase of £69 over those of the preceding year. Donations, always a fluctuating source of income, reached a total of £140, including a loan from the treasurer of £50. This compared in one way unfavourably with the previous year, when £230 was received, in addition to a loan from the hon. treasurer of £100, which had since been converted into a gift. Registration fees, including advertisements in the "Annual Register," now for the first time brought into the general account, amounted to £132—a small increase of £7 over those of the previous year. The "Nurses' Journal" showed a profit of £21, and this had been handed over to the general account. In addition to this a sum of £10—the outcome of nurses' contributions—had been paid out of the Journal's profits to the Helena Benevolent Fund, making a total surplus of £32. In "special accounts" there was an excess of expenditure over income of £15, £9 of which was accounted for by loss on the conversazione. The income had exceeded the expenditure by £71, towards which surplus the "Nurses' Journal" had contributed £21. The Helena Benevolent Fund had proved a material help to many nurses needing temporary assistance. Assistance had been granted to the extent of £21, leaving invested £79, and a balance in hand of £33. The Journal account showed a very healthy state, £173 having been received from advertisements, with £55 still owing. This compared with £81 paid for, and £150 owing for advertisements in the previous year. This satisfactory result was almost entirely due to the able and energetic editorship of Miss Leigh, the secretary. The net assets of the association after all liabilities had been discharged, stood at £289—a result which the hon. treasurer hoped would meet with the approval of the members.

Miss Entwistle asked if this would not be a convenient time to raise the question as to the appointment of an editor to the Journal, and on this being assented to stated her objection to such an appointment having been made without consulting the editorial committee or the general council.

Mr. Fardon, the medical hon. secretary, maintained that the matter had repeatedly been talked over by the editorial committee, and the step was taken with their approval; and also that the executive committee which had made the appointment was quite within its powers in doing so.

The Chairman held that no irregularity had occurred. The matter had been duly voted upon and carried by the executive committee, which, under the charter was empowered to "appoint such paid officers . . . as they may deem necessary, and . . . prescribe their respective duties."

Mr. Gant said that as the question of legality was settled the matter resolved itself into one of good judgment, and paid a warm tribute to the great improvement that had taken place in the literary matter of the Journal.

The Chairman wished it to be understood that although a lay editor had been appointed, the columns of the Journal were just as open to nurses as had always been the case.

The adoption of the hon. treasurer's report was then carried.

The report of the Executive Committee was then read and adopted. It stated that since the last meeting of the Council 41 nurses had been registered, 32 had been elected members of the Corporation, 1 had died, and 13 had withdrawn. The names were reported of the retiring members of the General Council and those proposed for nomination to the vacant seats at the annual meeting to be held on June 3rd. It was announced that in the interest of nurse members the names of those holding special qualifications in midwifery had been published as an appendix to the Roll of Members. The eleventh annual conversazione was held in February, and was well attended. Special thanks were accorded to those who had delivered sessional lectures, of whose kindness members had shown warm appreciation. Added to the report was the first list of ladies appointed to act as lady consuls.

The next business was the question of the removal from the Roll of Members of the names of two nurses on the ground of moral delinquency, and after some discussion the resolutions effecting this were carried.

The confirmation of a number of regulations with regard to meetings of the general council and the benevolent fund, duly passed at the meeting of the general council on January 1st, was then carried, as was also the adoption of new regulations relating to the badge of the association and the lady consuls.

These regulations were as follows:—

### The Badge of the Association.

1. All members shall be entitled to wear the badge of the corporation.
2. Any person who ceases to be a member shall return her badge to the secretary.
3. On the death of a member the badge shall be returned to the secretary by the representatives of the deceased.
4. The badge shall be made in bronze for members; in silver for past and present members of the general council and lady consuls; and in gold for the president, and for such vice-presidents of Royal or Princely family as may express themselves willing to receive it.
5. The price of the bronze badge shall be three shillings and that of the silver half a guinea.
6. The name and roll number of the member to whom the badge is granted shall be engraved at the back of the badge.
7. The badge shall be worn upon the right breast, suspended from a clasp.
8. Those members who desire to receive the badge shall make application for it on a special form, which can be obtained from the secretary.

### Lady Consuls.

1. The association shall be represented in various localities by lady consuls, who shall themselves be members of the corporation, and shall reside in the locality to which they are appointed.
2. Lady consuls shall be elected by the Executive Committee, and shall be subject to re-election in January of each year.
3. The duties of lady consuls shall be such as the Executive Committee may from time to time determine.
4. Members elected to serve as lady consuls shall be entitled, so long as they retain the office, to wear the silver badge of the association, together with a ribbon and clasp bearing the words "Lady Consul."

A list of institutions was then drawn up of which the matron or superintendent of nurses is entitled to be an *ex officio* member of the council, provided she be a member of the association and willing to act. These were selected on the principle that those institutions were entitled to such representation provided they had a medical school, contained 200 beds, and adopted the three years' system. It was pointed out that the list was not necessarily complete, since, though it contained none unworthy, some might have been omitted which ought to be there.

This concluded the business of the meeting.

### Echoes from the Outside World.

AN OPEN LETTER TO A HOSPITAL NURSE.

Feeling sure that you will be paying a visit to the Royal Academy as soon as you can, I managed to get an hour there on Monday so as to be able to give you some sort of idea what to look out for. First of all, a note of warning. Don't go expecting great things, because assuredly if you do you will be doomed to disappointment. Before May dawned I had heard of many artists being refused this season who had been accepted for years, and of those who had been accepted being "crowded out" for want of space, amongst others the artist policeman, Jones, of Leeds. So I had begun, rather naturally, to fancy the show of 1899 would be superlatively good, whereas it is decidedly mediocre, if not below the average. There is very little of a striking character. The loyal subjects of Her Majesty hover round the three Jubilee pictures, and compare notes as to which they prefer. All represent the Queen at St. Paul's in the State coach with the cream-coloured ponies. Mr. Gow's picture holds the place of honour in the second room, and has been painted for the Corporation of London. The second has been painted for the Queen by Mr. J. Charlt n. The third is the work of an Italian, Signor Gennaro 'Amato, and does not seem to have been ordered by any one in particular. Perhaps for that very reason it is generally considered the most satisfactory. The subject is treated with greater freedom, and the effect of the sunlight is more truthfully depicted. There are few paintings which attract a crowd, but foremost amongst them is "Love the Conqueror," by Mr. Byam Shaw, a young artist who has hitherto been remarkable principally for the strangeness of his pictures, which have excited more comment than admiration. But his large canvas in the tenth gallery will, this year, undoubtedly attract a large measure of attention. The scene is an allegorical one. Love, a pretty winged child, clad in golden armour and seated upon a black horse trapped with red, watches a mighty procession of his victims pass before him. Foremost in the band is Venus, bound with yellow silken scarves, the ends of which are held by merry Cupids. Behind come poets and painters, historical personages, and mythical characters. One recognises easily Shakespeare and Mary Queen of Scots, Michael Angelo and Henry VIII., Dante and Titania, but it would not be difficult for a fairly well-read person to identify nearly everybody who passes in that motley crowd, which is broken now and then by more Cupids holding their captives in silken chains. "The Lonely Life," by Hugh Rivière, will appeal to all women, the sadness of the sweet face of the "Princess, King-descended, decked with jewels," longing to be a peasant girl, untrammelled and free, being especially pathetic. Mr. Abbey's pictures are wonderful as works of art, but very hard to understand. Looking at "Who is Sylvia? what is she that all the swains commend her?" I felt a sneaking sympathy with the Bohemian young man behind me who remarked to his friend, "Who indeed? Anyhow, I don't admire their taste." "The Battle of the Nile," by Mr. Wyllie, gives a splendid idea of the havoc wrought amongst the ships by the opposing foes, and it has been purchased by the trustees of the Chantrey Bequest. Do not forget to look for the two pictures by Mr. George Clausen, and for an amusing picture called "An Early Rehearsal," by Mr. Markham Skipworth. Two clever and natural portraits (of which particular branch of art there seems less than usual in the exhibition, it being decidedly a landscape year) are "Stella," by Louisa Starr, and "The End of the Story," by Arthur P. Burton. I cannot say that there is anything of special interest to nurses.

---

Going to the Academy we were stopped for a long time to allow the Labour Demonstrators to pass on their way to Hyde Park. I had never seen their May Day procession before, and it much impressed me. I could not help reflecting that if the ill-clad, unhealthy, long-visaged men who marched by me were good specimens of the sort of people who gather under the Socialist banner, decidedly Socialism does not attract the cream of our working men. The women were even worse, hanging on to each other's arms, as if partly for support, slipshod, be-feathered, with uncurled fringes half hiding their eyes, and the same depressed, dissatisfied expression I had noticed on the faces of the men. The little children supplied the only bit of brightness. The fact of wearing a red paper rose, eating oranges galore, and sometimes in addition of riding in a brake, was enough joy for them. Remembering the trial scene in "The Only Way," which admittedly gives a very fair representation of a gathering of "citizens" at the time of the French Revolution, I tried to imagine for a moment what some of the faces in that crowd might become if inflamed by rage and vengeance against the capitalists and aristocrats of England. And as I thought I trembled.

---

At one of the handsome new suburban theatres the other night a little incident happened which caused an immense amount of amusement to all the audience, with the exception of a single person. The play, which was a tragedy, had progressed as far as the second act, and the time had nearly arrived for the curtain to go up for the last scene, when suddenly the house became convulsed with laughter. Strangely enough, nearly everybody seemed to see the joke at the same moment, and those few who did not supplied an additional element of fun by the comic way they twisted themselves into all kinds of attitudes, and craned their necks, to get a view of what was going on. A lady in the front row of the upper seats had been leaning forward to have a peep at the folks below, and her front fringe, being insecurely fastened, had become detached from the rest of her hair and had gently fluttered down on to the heads of the people underneath. Being so light in weight, the descent was particularly slow, and the mass of fair curls fluttered here and there as the breeze swayed it, till finally, like a feather, it sank gently down on to the top of an old gentleman's head. Here for a second it reposed. But the frightened look of the old gentleman at finding something soft unexpectedly alighting, and the indignant sweep of the hand which removed the hirsute appendage was irresistibly funny. As to the unfortunate lady who had sustained such an awkward loss, I could not see her; but I am told that as soon as she realised what had happened she seized her hat, crammed it well down over her poor forehead, and fled, and that a fair "bang," beautifully curled, now lies at the box office of that theatre awaiting an owner.

---

The Americans seem to have a strange idea of what is suitable for a Sunday school entertainment. The latest development has been a mock wedding in a Congregational church, "played" by children. The "bride," a child of seven, was attired in the orthodox white satin dress, with a long train, and wore a veil and orange blossoms. The "bridegroom," aged eight, was—according to American custom—in evening dress, the "clergyman" being nine. "Bridesmaids," best man, and the usual guests were included in the company. The march from "Lohengrin" was played by the organist, and a "bridal" feast was afterwards given in the Sunday schoolroom. The adult congregation who came as spectators are reported to have "enjoyed the scene" as much as the children. All I can say is that if such was the fact they ought to have been ashamed of themselves, and the sooner Sunday school entertainments of such a type are done away with the better for the American children.

### Everybody's Opinion.

[Correspondence on all subjects is invited, but we cannot in any way be responsible for the opinions expressed by our correspondents. No communication can be entertained if the name and address of the correspondent is not given, as a guarantee of good faith but not necessarily for publication, or unless one side of the paper only is written on.]

### THE ROYAL NATIONAL PENSION FUND.

"Policy Holder 2,039" suggests that a Home should be provided by the R.N.P. Fund, where the members, on attaining their pensions, could take up their abode, their savings covering their expenses. By clubbing together in this way, she urges, each one's money would procure them greater comfort in their old age. It might be arranged so that the members should have the option of either receiving their pensions or entering the Home.

### THE POSITION OF A NURSE IN A POOR-LAW INFIRMARY.

"Another Superintendent of Nurses" writes: Before coming to the pith of my letter may I just say how much I appreciate The Hospital and "Mirror." I look forward every Friday to the newsboy's arrival, and always try to get a quiet hour in which to "read, mark, and inwardly digest" its contents. It has seemed to me for some long time that the difficulties attending a superintendent of nurses' position in a Poor Law infirmary are not in the least degree ameliorated by the discussions constantly going on in the different papers of the day. For my own part I must confess my lines have fallen in very pleasant places, for the matron here is a well-educated and very capable woman, and has never in any way interfered in my work. Would that there were more such among the matrons! Of course, all superintendent nurses must more or less thank Miss C. J. Wood for taking up the cudgels for them, but one cannot help feeling that the result is so far entirely unsatisfactory. Why should not the superintendent nurses do something for themselves? Send up a petition to the Local Government Board signed by all of them, asking those gentlemen to deal with the affair for them. I am sure they have the welfare of the nurses at heart, and would do their best for them. I don't know whether this suggestion is feasible, but I thought if so you, at any rate, were the person above all others to bring the matter forward.

### A DEARTH OF TRAINED NURSES.

"Bluebell" writes from Yorkshire: In answer to the letter appearing in the "Mirror" from a "Superintendent of Nurses," may I suggest two reasons why trained nurses do not care to join nursing homes—first, they are not offered sufficient remuneration for their services; second, there are so many hard and fast rules to be kept. With regard to my first reason, how is it that so little salary is offered in proportion to the earnings of the nurse? I know of several cases where the matron of an institution was charging from one and a half to two guineas weekly for the nurse's services, keeping her constantly occupied, with scarcely any rest between her "cases," while the nurse herself was receiving only a paltry salary of £25 and uniform. I believe in many homes £30 per annum is given, but in very few is it over £35. Surely the nurse is entitled to a larger share of her earnings than this? If the working expenses of a nurses' home are so considerable, is it any wonder that nurses should prefer to "form small establishments and take their own fees," or else join co-operative institutions? I would suggest that nurses should be paid in certain fixed proportions according to their earnings, and also that the institutions should be made more home-like. Coming in from a hard case a nurse naturally likes to have a little liberty and be able to come in and go out as she pleases, or to have a little extra rest in bed instead of having to get up to eight o'clock breakfast. If more consideration could be shown in these things I think there would be no lack of candidates for the nursing institutions.

"A Nurse" writes: I have been in one of the homes for trained nurses, and I think I shall be a long time before I am persuaded to join another one. Certainly, it was not an old-established one. If I rightly remember I think it had been started about two years previous to my joining it. I had an interview with the matron before I became engaged, saw the rules, &c., and everything seemed to be quite right. Before I had finished my first case the matron wrote to me and said they were removing into a smaller house, but that there was heaps of room, &c., and if there happened to be more nurses than she could provide with sleeping accommodation she had engaged beds elsewhere. It was quite six months before I went into the home to sleep, then I had work in the town, and for ten weeks I was in every night. What I went through in those ten weeks I shall never forget. I was subjected to the grossest insults from the matron's husband, who posed as a gentleman, and it got so unbearable that I left within an hour's notice, and I have since had to resort to extreme measures to get my salary. When I say that I am but one of many who have had a similar experience in the same home, many of whom represent different counties, and who naturally repeat their experiences to others, is it to be wondered at that nurses are getting shy of joining these homes? I do not wish to lower the standard of nurses' homes in the least, especially the old-established ones, many of which, I believe, are very satisfactory in all points. But until such places as this I speak of are condemned and stamped out of existence, I see no hope of a better state of things, but rather think there will be still more difficulty in getting trained nurses to join these institutions.

"C.," referring to the letter of "A Superintendent of Nurses," writes: I do not know how these nursing institutions are managed now, but when I began private nursing some seven or eight years ago I know what we nurses thought of them then. No fully-trained, certificated nurse would have dreamt of joining them, and the fault lay with some of the old-established institutions. I am acquainted with more than one which took women of only a year's, or even a few months' training, and sent them out to the public as "fully-trained nurses," charging one and a half and two guineas weekly, whilst the nurses received a yearly salary, beginning with £18 or £20, with uniform. Someone, not the nurses, took the profits, and it is a well-known fact that the owner of one of the old establishments made a small fortune. Again, some of them were started by people not in the nursing profession at all, and were therefore run merely as business speculations. As an older member of the profession I much regret that they are on the increase. Any nurse now thinks she may start a nursing co-operation by collecting a few nurses together and advertising them as taking their own fees, whereas a properly-constituted society must have a sufficient number of members and suitable people to organise and work so that it may become a success professionally and financially. I am sure that every sensible woman recognises the advantages of union and co-operation, as shown by the success of the two well-known London associations, which are so managed that they are the just pride of their members and their chiefs.

### The Nurses' Bookshelf.

The Art Portfolio. (Simpkin, Marshall, and Co. Monthly. Price 1s.)

We have received a copy of Part II. of this interesting publication, which contains four large and beautifully-executed photogravures of "The Vigil," by John Pettie; "Entrance to the Zuyder Zee," by Clarkson Stanfield; "The Frugal Meal," by J. F. Herring; and "Noonday Rest," by John Linnell. Each month four celebrated pictures are reproduced, and it is pleasing to learn that the work is executed in England. Subscribers will find themselves possessed of a charming collection of reproductions of the works of our best artists. Each one is worthy a frame.

### Appointments.

WAKEFIELD CITY FEVER HOSPITAL.—Miss Edith Thomas has recently been elected Matron. She was trained at the County Hospital, Durham, and then became charge nurse for two years to the female surgical and accident ward at the Bradford Royal Infirmary. She has since been charge nurse for two and a-half years to children's ward and the Male medical ward, also to the abdominal wards and operating theatre at the Huddersfield General Infirmary. Subsequently she became charge nurse for another two and a half years to the male accident ward at the Beckett Hospital, Barnsley.

ST. GILES', CAMBERWELL, PARISH INFIRMARY.—On the 26th ultimo Miss Mary E. Mead was appointed Assistant Matron and Superintendent Nurse. She was trained at the London Hospital, where she was afterwards staff nurse and sister for five years altogether. From July, 1895, to December, 1896, she was sister at the Civil Hospital, Hongkong, and matron of the General Hospital, Yokohama, from May, 1898, to January, 1899.

ROCHDALE UNION WORKHOUSE INFIRMARY, DEARNLEY—On April 27th Miss Maude M. Wray was elected Superintendent. She was trained at the Liverpool Royal Infirmary, and she has held the appointment of charge nurse at Fir Vale Infirmary, Sheffield, and that of night superintendent at the City of London Infirmary, Bow.

DISTRICT COTTAGE HOSPITAL, ULVERSTON.—On April 25th Miss Agnes Tyson was appointed Matron. She was trained in the North Staffordshire Infirmary, and her previous appointments were at the Borough Hospital, Hull, and the Queen Victoria Jubilee Institute of Nurses.

CHESTER LADIES' CHARITY INSTITUTE.—On the 29th ultimo Miss Hazlen Davies was chosen Matron.

### Minor Appointments.

CAMBERWELL INFIRMARY.—Miss Annie Wood has been elected Head Nurse. She was trained at the Sheffield General Hospital, and has since held appointments as charge nurse to the Rochdale Infirmary from January to July, 1893, and at the North London Hospital and South Western Hospital from September, 1893, to May, 1898.

WOLVERHAMPTON AND STAFFORDSHIRE GENERAL HOSPITAL.—Miss Evelyn M. Dane was appointed Night Superintendent on April 25th. She was trained in the Western Infirmary, Glasgow, and has been charge nurse at Fountain Hospital, Tooting.

PARISH WORKHOUSE, ST. PANCRAS.—Last week, Miss Matilda Lipscombe was appointed Charge Nurse. She was trained at Poplar and Stepney, and her previous appointments were at Islington Infirmary and West Ham Cottage Hospital.

### To Nurses.

IN order to increase and vary the interest in the *Mirror*, we invite contributions from any of our readers in the form of either a paragraph, or information, and will pay a minimum of 5s. for each contribution. All payments are made at the beginning of each quarter, *i.e.* January 1st, April 1st, July 1st, and October 1st.

### Wants and Workers.

"A DISTRICT NURSE" wishes to know if any readers of "The Nursing Mirror" have a Merlin chair to dispose of. It is for a young married woman, the wife of a railway porter, helpless through rheumatism. £1 or 30s. could be managed, but not more. Address Nurse Bird, 6, Lonsdale Street, Stoke-on-Trent.

### For Reading to the Sick.

ABIDE with us, for it is toward evening and the day is far spent.—*St. Luke* xxiv. 29.

My soul waiteth for the Lord more than they that watch for the morning; I say more than they that watch for the morning.—*Ps.* cxxx. 6.

Unto Thee lift I up mine eyes, O Thou that dwelleth in the heavens.—*Ps.* cxxiii. 1.

AT EVENTIDE IT SHALL BE LIGHT.

Lead, kindly light, amid the encircling gloom,
Lead Thou me on ;
The night is dark and I am far from home,
Lead Thou me on.
Keep Thou my feet ! I do not ask to see
The distant scene ; one step enough for me.

I was not ever thus, nor prayed that Thou
Should'st lead me on ;
I loved to choose and see my path, but now
Lead Thou me on.
I loved the garish day, and spite of fears,
Pride ruled my will ; remember not past years.

So long Thy power hath blest me, sure it still
Will lead me on,
O'er moor and fen or crag and torrent till
The night is gone ;
And with the morn those angel faces smile
That I have loved long since, and lost awhile.

—*Newman.*

**Reading.**

In order to a full perception of our need of God's Light we must remember how human reason has been darkened since Adam's fall, and that no earthly wisdom can suffice to guide us in the hidden paths of grace. God wills us to tread them in faith only, and so He only gives us just such light as we need for the present moment. It is not His will that we should see before us, or around us, but He never fails to grant such Light as makes it impossible for us to lose our way so long as we follow His leading.

You must continually seek Divine Light, ask for it on every occasion, great or small, undertaking nothing without it.

The Spirit of God cannot be fettered or subject to your control ; you must wait patiently, certain that He will never fail you in the hour of need.

In order to make a right use of Divine Light you must avoid as far as may be giving way to imagination and your own opinion, mistrusting your reason and judgment.

The best use of reason in spiritual matters is to offer it silently at the foot of the Cross. God makes Himself known chiefly to those who are lowly and childish in heart.

He cares nothing for profound learning or brilliant talents, save inasmuch as they are sanctified by being offered to Him. He would have us put aside all human knowledge, confessing that we know naught, save through Him.

All worship involves a perpetual confession that He is the Light and the Truth, we all darkness and falsehood. Let us then sing with David, "O grant me understanding that I may know Thy testimonies. Give me understanding and I shall live."—*From the "Hidden Life of the Soul."*

It is with man's soul as it is with nature ; the beginning of creation is—Light. Till the eye have vision the whole members are in bonds. Divine moment! When over the tempest-tossed soul, as once over the wild weltering chaos, it is spoken, Let there be Light.—*Carlyle.*

**Prayer.**

O, Holy Jesu, send down Thy heavenly Light into my darkened sin-laden heart. Give me strength amid the shadows to press on, in patience and humility, to strive valiantly, to hope fearlessly, and to pray ceaselessly for strength to bear the cross Thou hast laid upon me. Thou who hast known darkness and anguish of mind and body such as I can never know, in mercy visit my weary soul and give it Light and Peace until the day break and the shadows flee away. Amen.

### Travel Notes.

By Our Travelling Correspondent.

### XXI.—AN EASY TRIP FOR NURSES—THE BAY OF ST. MALO.

Supposing you have only a fortnight to spare, and a somewhat slender purse, you cannot do better than explore the Bay of St. Malo for your holiday.

### Choice Between Paramé and St. Servan.

Dinard is very expensive even out of the season, so you must not think of it as a residence, but by staying at Paramé or St. Servan you can enjoy all the pleasures of Dinard, such as bathing and the gaieties of the casino, without the heavy expense of residence there. St. Malo itself is not desirable as a residence owing to lack of air. It is an ancient fortified town, and the houses are so tall and the streets so uncommonly narrow that there is not sufficient ventilation. It affords endless subjects for the artist, but I should recommend living outside the walls. St. Servan, Paramé, and St. Malo are all connected by tram, and St. Servan and St. Malo by what is called the Pont Roulant, a singular little rolling bridge something resembling a bathing machine, which moves on slender legs some fifty feet long with small wheels attached across lines laid in the bed of the dock. All these three towns are also connected with Dinard by a steam ferry, which crosses in fifteen minutes every half-hour. Living may be had very cheaply both at Paramé and St. Servan, and each can claim some advantage. For my own taste I greatly prefer St. Servan, Paramé being purely and simply a seaside resort and overrun with children and nursemaids, but I must concede that the air is magnificent and pure, and that the hotels are reasonable and good. Paramé is a new place and lacks the dignity of St. Malo and St. Servan. For a short holiday, St. Servan is I think, preferable; it is much closer to the steam ferry for Dinard, it is a better starting point for the lovely expeditions up the Rance, it is better situated for the railway, and is only five or ten minutes' walk from St. Malo. If any of you think of making this trip, and will tell me how much you can spend, and what your special tastes are, I will advise you as to locality and hotels or pensions.—

### Residence at St. Servan.

I shall conclude that you have decided on St. Servan, then, and shall make that our headquarters in considering the locality, but first as to the journey.

### Cost of the Return Ticket.

From London to St. Malo and back is, first class, £2 12s., and, second, £1 19s. 6d. Second is quite agreeable, and there is nothing to object to at all. There is no railway journey on disembarking, and if you have but little luggage you can go round in the steam tram which starts from the Custom House, price 20 centimes. If you have much impedimenta you must take a carriage and make a bargain beforehand; the very agreeable drivers have a weakness for getting all they can. It is a great charm to the unaccustomed traveller, and one perhaps not very conversant with French, to feel that the toils of the journey are over and "tea" and rest looming near. The approach to St. Malo is impressive, the English boat anchors under the grand old ramparts and generally near to the massive Porte de Dinan. The singularity of this sea-girt town must strike even the least observant; in olden days the inhabitants were a species of Corsair, who came out from their island nest and robbed and pillaged right and left. The cathedral is nothing remarkable, but its position is quaint and unusual. The cathedral of St. Servan, though modern, is much finer; there is a very beautiful group of statuary in the lady chapel, and the carving of the pulpit is well worthy of notice. On the quay, where you take the ferry for Dinard, is a singular interesting trefoil tower called the Solidor; until very lately it was used as a political prison.

### Excursions in the Vicinity.

These are endless, and want of space will prevent my telling you even of half of them. I will therefore mention the most important. First, then, to Dinan, a small town about 18 miles inland. The most direct way to reach it is to cross to Dinard and take the train. The station at Dinard very successfully conceals itself from view, and is only discovered after much research. It is, however, only ten minutes' walk. In spite of hordes of tourists, the connecting line with Dinard, and the fact that it is a garrison town Dinan still retains its mediæval character; but few new buildings have been added, and gay uniforms look out of

Looking up the Jersual.

wonderful fifteenth century windows, and swords and spurs clank up and down pitched streets, the artistic charms of which can hardly be surpassed. Foremost among these is

### The Rue Jersual.

This ancient approach to the town from the little port on the Rance is almost a pure specimen of the domestic architecture of the sixteenth century; I send you a sketch taken from the riverside of the mighty gateway looking up this wonderful street; it required some courage to sit there and sketch, for the perfumes are not those of Araby. Another day I think I must take you to Dinan again, for there is material there for many holidays.

### Voyage up the Rance.

It would be well (according to the tide) to go to or return from Dinan by the Rance. Steamers go twice a day, and the little voyage is delightful; the banks of the river are in many places very wild and precipitous and richly wooded; and mountainous hills descend to the water's edge. Of the many lovely excursions about the banks I must speak another day.

LA GARAYE—CONINNAIS.

It is better really to go to La Garaye when staying in Dinan, but it is quite a practicable excursion from St. Servan, and can be visited the same day as Coninnais. Most people have read Mrs. Norton's beautiful poem concerning it. Before the Reign of Terror, the last Count of La Garaye, with his noble and gentle wife, established a species of hospital for the surrounding poor, whom they nursed personally, acquiring considerable surgical skill, especially in eye cases. In 1793 the mob reduced the building to its present ruined condition. Coninnais is preserved in good condition and is inhabited; it is a very picturesque building as seen from the old coach road. Next week I will tell you about Mont St. Michel, an enchanting spot which cannot be dismissed in a few words. It is quite possible to explore it in a long day's excursion, but it is fatiguing, and naturally one only sees it thus in a perfunctory manner.

### TRAVEL NOTES AND QUERIES.

RULES IN REGARD TO CORRESPONDENCE FOR THIS SECTION.—All questioners must use a pseudonym for publication, but the communication must also bear the writer's own name and address as well, which will be regarded as confidential. All such communications to be addressed "Travel Editor, 'Nursing Mirror,' 28, Southampton Street, Strand." No charge will be made for inserting and answering questions in the inquiry column, and all will be answered in rotation as space permits. If an answer by letter is required, a stamped and addressed envelope must be enclosed, together with 2s. 6d., which fee will be devoted to the objects of the "Hospital Convalescent Fund." Any inquiries reaching the office after Monday cannot be answered in "The Mirror" of the current week.

SPAIN FOR ECONOMY (Ruby).—Yes, living is very cheap now in Spain, but you could not live in apartments unless you have a fair knowledge of the language. French does not help you much, except in Madrid. You do not tell me where in Spain you would like to settle. Northern Spain is bitterly cold, except such sheltered spots as St. Sebastian, which is anything but cheap.

CYCLING (Wheelwoman).—You must belong to the C.T.C. if you wish to take your machine. If not, you will be put to endless expense and annoyance.

PYRENEES (The Mountaineer).—Climbing is difficult in the Pyrenees because there is but little snow and ice, and no possibility of cutting steps. Quite unsuitable for women, but if you are content with mountain walking it is an ideal country for it. You might go up into the mountains at the end of May.

SWISS TOUR (Venice).—Take warm, light clothing, but it should certainly be wool. (2) High lacing boots, with square heels. A waterproof cape is a necessity. The mountain storms come on very suddenly. In going expeditions strap it round your waist, and you are prepared for emergencies. Trimmed hats quite out of place.

*(For Travel Advertisements see Page xvi.)*

### Dr. Lunn's Tours.

THESE fascinating and inexpensive tours begin this week, and Dr. Lunn has catered for all tastes. For those whose time is very limited, and who are, perhaps, inexperienced in foreign travel, they are a real boon. A nineteen days' cruise to Norway, the cost of which is only sixteen guineas, starts on June 17th. A still cheaper one, lasting thirteen days, leaves England on July 8th, and a similar one on July 22nd. For those who take their holidays later a Norwegian tour is fixed for August 5th; thirteen days' cruise for eleven guineas, and a twenty-five days' cruise for twenty-one guineas. Then for Switzerland there are enchanting little tours of nine days for six guineas, and longer ones for proportionate sums, starting twice a week through May, June, July, August, and September. A very nice one of eighteen days for £13 8s. is as far as Chamounix. This is arranged for May 30th, June 27th, and August 4th, and some as late as September. Dr. Lunn has also arranged for Scotch tours this year, which will be a novelty and a great boon to timid travellers who fear to venture thither on account of the heavy expenses incurred by independent tourists. The cost of these tours is £11 4s., which includes ten days' accommodation at hotels. They will start on eight different dates; those in the height of the season, July and September, will be a little more expensive.

### Notes and Queries.

**The contents of the Editor's Letter-box have now reached such unwieldy proportions that it has become necessary to establish a hard and fast rule regarding Answers to Correspondents. In future, all questions requiring replies will continue to be answered in this column without any fee. If an answer is required by letter, a fee of half-a-crown must be enclosed with the note containing the enquiry. We are always pleased to help our numerous correspondents to the fullest extent, and we can trust them to sympathise in the overwhelming amount of writing which makes the new rules a necessity.**

**Every communication must be accompanied by the writer's name and address, otherwise it will receive no attention.**

*Emigration.*

(53) Could you give me information respecting emigration societies for girls, and whom to write to in connection with such?—*M. A. S.*

The United British Women's Emigration Society, the Imperial Institute, W. Write to the Secretary.

*Treatment by Oxygen.*

(54) An adult lady friend has suffered from recurrent acute headache for five years. Numerous specialists and others have been consulted, but their respective treatments have proved unavailing. Would the "oxygen cure" be beneficial? Is it practicable in the private house? By what means is it undergone?—*Haus.*

We cannot prescribe. Oxygen can, of course, be administered in a private house. It would be more accurate to speak of "treatment by oxygen" than of "oxygen cure."

*Training.*

(55) Kindly tell me what position I should be able to take after two years' training in a chest hospital and one year in a good general hospital? Would a nurse with this training have any difficulty in obtaining a post as sister in a general or children's hospital?—*Perplexed One.*

Our advertisement columns will show you what posts you may hope to obtain with the training you mention. But a three years' certificate is a qualification almost invariably required from candidates for a sister's post in a general hospital. You certainly would not obtain any responsible appointment in a children's hospital without experience in the nursing of children. Why not continue training at the general hospital where you have been for a year, and so obtain a certificate which will be of substantial value in the future?

*Home Wanted.*

(56) Can you tell me of a home where a gentleman of very limited means suffering from locomotor ataxy could be received? He is able to get about, but requires care. A small sum could be paid.—*Nurse Agnes.*

You will find full particulars given of homes in "Hospitals and Charities," published by the Scientific Press, 28, Southampton Street, Strand, and can there see for yourself which best suits the case in point. We acknowledge, with many thanks, P.O. for 2s. for Convalescent Fund.

*Advice Wanted.*

(57) I am a nurse-companion with a good deal of leisure. Do you think I could attend the lectures at a neighbouring hospital or find a little work to do in connection with it, perhaps one day in the week? I would gladly pay for the benefit of the instruction if I might attend the lectures.—*Nurse B.*

Write to or call on the matron of the cottage hospital you mention and put your question to her. Very possibly some arrangement of the kind might be managed. Perhaps you might find your help would be valued by a district nurse in your neighbourhood. There is always plenty to be done, and willing hands are generally welcome.

*Medical Education.*

(58) Will you be kind enough to tell me where to find the names of hospitals and dates of entrance and leaving by English doctors?—*Mercury.*

What can you mean? "The dates of entrance and leaving" at a medical school are not regulated by hard and fast rules. The summer session begins in May, the winter session in October, and the length of time occupied in qualifying for degrees depends upon the brains and working capacity of each student. The fullest particulars of the medical schools are to be found in the Medical Directory. For further information write direct to the various schools.

*Southern Europe.*

(59) Would you kindly give me the address of any private nursing institute either in Italy or the South of France?—*Catherine.*

The Hollond Institute, 1, Tavistock Chambers, Bloomsbury, has numerous branches abroad.

*Home Nursing.*

(60) Could you recommend a good, practical book on home nursing?—*J. C.*

One of the following might suit you, and the Scientific Press, London, would, at your request, procure and forward it for you: "Home Nursing," by R. A. Neuman, 1s. 6d.; "Handbook of Nursing for the Home," by F. Staacpoole, 1s. 6d.; or "Home Nursing," by Dr. Weatherley, 1s.

*Musical Instruments.*

(61) Referring to "Nurse Nina's" question (No. 48) as to a small musical instrument easy to learn, a nurse from Reading kindly writes to recommend a Wheatstone English concertina. It is very portable, not difficult to master, and she has found it much liked by her patients when softly played.

*Anonymous Inquirer.*

X. Y. Z.—See notice at the head of this column. No notice can be taken of any letter which is not accompanied by writer's name and address, not for publication, but for the Editor's information only.

### ANSWERS REQUESTED.

*County Hospital, Huntingdon.*

The Manager has received a communication, headed as above, from a nurse who does not sign her name. Will she kindly send it that her request may be attended to.

## Figures and Tables

**Figure f1:**